# Experimental Study on Secondary Anchorage Bond Performance of Residual Stress after Corrosion Fracture at Ends of Prestressed Steel Strands

**DOI:** 10.3390/ma16237441

**Published:** 2023-11-29

**Authors:** Rihua Yang, Yiming Yang, Xuhui Zhang, Xinzhong Wang

**Affiliations:** 1 School of Civil Engineering, Hunan City University, Yiyang 413000, China; 2 Hunan Engineering Research Center of Development and Application of Ceramsite Concrete Technology, Hunan City University, Yiyang 413000, China; 3 School of Civil Engineering and Mechanics, Xiangtan University, Xiangtan 411105, China

**Keywords:** bond performance, bridge engineering, corrosion fracture, numerical simulation, secondary anchorage

## Abstract

In order to explore the secondary bond anchorage performance between prestressed tendons and concrete after the fracture of steel strands in post-tensioned, prestressed concrete (PPC) beams, a total of seven post-tensioned, prestressed concrete specimens with a size of 3 × 7ϕ15.2 mm were constructed firstly, and the steel strands at the anchorage end were subjected to corrosion fracture. Then, the pull-out test of the specimens was conducted to explore the secondary anchorage bond mechanism of the residual stress of prestressed tendons experiencing local fracture. Moreover, the influences of factors such as the embedded length, release-tensioning speed, concrete strength, and stirrup configuration on anchorage bond performance were analyzed. Finally, the test results were further verified via finite element analysis. The results show that the failure of pull-out specimens under different parameters can be divided into two types: bond anchorage failure induced by the entire pull-out of steel strands and material failure triggered by the rupture of steel strands. The bond anchorage failure mechanism between steel strands and the concrete was revealed by combining the failure characteristics and pull-out load–slippage relation curves. The bond strength between prestressed steel strands and concrete can be enhanced by increasing the embedded length of steel strands, elevating the concrete strength grade, and enlarging the diameter of stirrups so that the specimens are turned from bond anchorage failure into material failure.

## 1. Introduction

The concept of stress transfer and anchorage, which derives from pre-tensioned specimens, refers to the process of stress increases in prestressed tendons at the beam end from zero to the effective stress and ultimate stress [1]. The pre-tensioned specimens complete the prestress transfer and anchorage through the bonding between prestressed tendons and concrete, and the successful bonding between them is the basis for the normal service of pre-tensioned members. Therefore, it is necessary to study bond anchorage performance between steel strands and the concrete of pre-tensioned concrete members. The relevant research results can also provide a scientific basis for the safety assessment and maintenance decision-making processes of bridges in the future [2,3,4].

It is generally believed that the bonding force between steel strands and concrete is composed of adhesion, mechanical interlock, and friction [5]. The bond anchorage performance of steel strands is similar to that of plain steel bars in the early loading stage, i.e., the bonding force is mainly provided by adhesion. In the later loading stage, mechanical interlock plays a dominant role since the twisting process is adopted for steel strands, and slippage is accompanied by the rotation along the twisting direction [6,7,8]. The bond performance between steel strands and concrete is correlated with concrete strength, the thickness of the concrete cover, the stirrup ratio, and the shape of prestressed tendons [9,10,11]. In some studies, bond stress–slippage models have been proposed through experimentation and theoretical analysis. As a result, the nonuniform distribution characteristics of bond stress along the embedded length have been determined [12,13]. With an increase in concrete compressive strength, the average bond stress increases while the anchorage length decreases [14,15]. Because stirrups can confine the development of splitting cracks, the slip amount is greater when anchorage fails. In other words, with an increase in the stirrup ratio, both the bond strength and ultimate slip amount increase. To avoid the failure of the structure due to anchorage failure, many national codes have given calculation formulas for the anchorage length of flexural specimens [1,16,17]. However, the existing research results show that the calculation of the anchorage length given in the codes is partially conservative and safe [9,18,19].

For bonded post-tensioned, prestressed concrete (PPC) members, the prestress is transferred through the anchorage in the prestress application stage, and the anchorage bonding is completed through the bonding between prestressed tendons and concrete in the working stage [20]. Due to construction defects, however, PPC specimens may experience insufficient grouting, so that the structure develops from fully bonded prestressed concrete to partially bonded prestressed concrete or even unbonded prestressed concrete. The structure can still work normally without any bond failure thanks to the end anchorage. In practical construction, due to construction defects such as the untimely closure of the anchorage end or cavities in the concrete at the anchor sealing position, corrosion occurs in the end anchorage zone with the extension of the service time, which leads to anchorage failure [21,22,23]. It is considered that, after the anchorage failure of the PC specimens, which is similar to pre-tensioned specimens, the residual prestress can be transferred and anchored for the second time through the bond with concrete [24,25]. Its mechanism is similar to that of pre-tensioned tendons but there are also differences. On the one hand, the prestressed tendons of post-tensioned specimens are mostly placed in the specimens in the form of multiple steel strands and the prestressed tendons interact with each other [26]; on the other hand, the corrosion process of prestressed tendons is slow, which is different from the rapid release-tensioning of pre-tensioned tendons. Rapid release-tensioning increases the initial damage of concrete and reduces the bond stiffness, which affects the secondary anchorage bond performance of the residual stress.

The secondary transfer and anchorage of residual stress after the stress fracture of prestressed tendons have been preliminarily studied [25]. In this study, the flexural performance of the specimens after the local fracture of a single steel strand was explored. The results show that the flexural bearing capacity of the specimens experiencing end anchorage failure only declined by 7.2% [24]. The calculation method for the bonding length of a single steel strand after end fracture was obtained by setting ribbed pre-embedded pipelines. However, corrugated pipes are usually used in practical projects, which is obviously not consistent with a real-world situation [25]. At present, the bond performance of tendons and concrete after the anchorage failure at the end of post-tensioned specimens has not been systematically investigated.

Therefore, bond performance between the steel strands and concrete after the end anchorage failure of the 3 × 7ϕ15.2 mm post-tensioned, prestressed concrete specimens is studied, and the bond mechanism and bond failure mode are discussed through pull-out tests and simulation analysis. Then, the key parameters influencing bond performance are captured. Finally, a finite element modeling method is proposed, and its rationality is verified based on experimental results.

## 2. Test Introduction

### 2.1. Specimen Design

In this experiment, a total of seven post-tensioned PC specimens were designed and manufactured, numbered S1–S7 in turn. Considering that the web width at the end of a small box girder is usually about 300 mm, the specimen width was also set to this value in this experiment. The specimen height was determined by considering both the height–width ratio and experimental economy. The cross-sectional dimensions of all pull-out specimens were identical, being 300 mm × 500 mm. In addition, for the actual prestressed concrete box girder, the concrete strength grades are usually C40 and C50, and the concrete strength grade of some structures also reaches C60. As a result, the effect of these three strength levels on the test results is also considered. Similarly, three commonly used stirrup diameters of 8, 10, and 12 mm are used for the comparative analysis. Moreover, the anchorage length of prestressed tendons that were estimated according to the specification of GB50010-2020 [16] should be less than 1300 mm, and only two types of specimens with lengths of 1000 mm and 1250 mm are designed here. See Table 1 for the detailed dimensions of the specimens.

**Figure 1:**
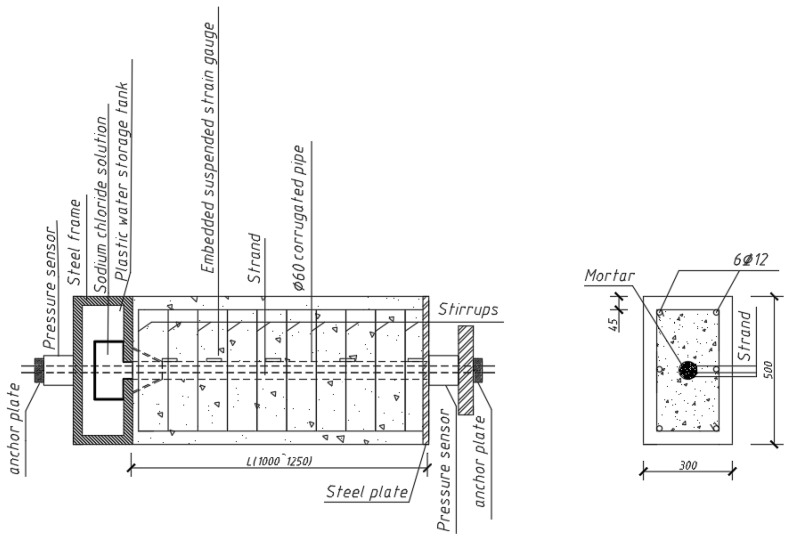
Dimensions and reinforcement of S1–S7 specimens (unit: mm).

A corrugated pipe with a diameter of 60 mm was reserved in the center of each specimen, seven-wire, twisted-steel strands (3 × 7ϕ15.2 mm) were designed in the corrugated pipe, and eight Φ12 mm HRB400 deformed steel bars were longitudinally arranged outside the corrugated pipe; stirrups were arranged around the longitudinal reinforcement at a spacing of 100 mm. In order to study the influences of specimen length, concrete strength, and stirrup ratio on the stress transfer and anchorage performance of fractured prestressed tendons, two bond length values (1000 and 1250 mm), three concrete strength grades (C40, C50, and C60), and three stirrup configurations (Φ8@100 mm, Φ10@100 mm, and Φ12@100 mm) were designed for the test specimens. The stirrups were HPB300 plain steel bars. The reinforcement layout of each specimen is displayed in Figure 1.

The tension control stress of steel strands is 1395 MPa, and the measured yield strength is 1810 MPa. The material properties of steel strands and ordinary steel bars are listed in Table 2. The concrete was composed of 42.5# ordinary Portland cement, natural river sand, graded aggregate, and laboratory tap water mixed together. The tap water in the laboratory was used as mixing water. Commercial cement mortar with the same grade as concrete was used as grout. Standard cubic blocks were cast using the same batch of concrete and maintained together with the test specimens. The average compressive strength of the concrete of the C50, C40, and C60 specimens at 28 d was 53.5, 42.6, and 63.3 MPa, respectively.

### 2.2. Tensioning of Prestressed Tendons and Effective Prestress

During the tensioning process, the tensioning force was monitored with the pressure sensor, and, meanwhile, the elongation of the steel strands was recorded using a dial indicator. At 48 h after tensioning, the reading of the pressure sensor was obtained, and the effective prestress applied by the member was measured, as seen in Table 3.

### 2.3. Stress Release of Anchorage Parts at the End of Prestressed Tendons

**Figure 2:**
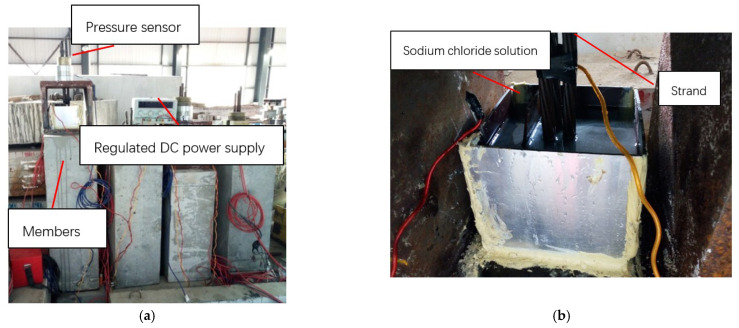
Corrosion position of test members: (**a**) corrosion process of specimen; (**b**) detailed structure of corrosion groove.

**Figure 3:**
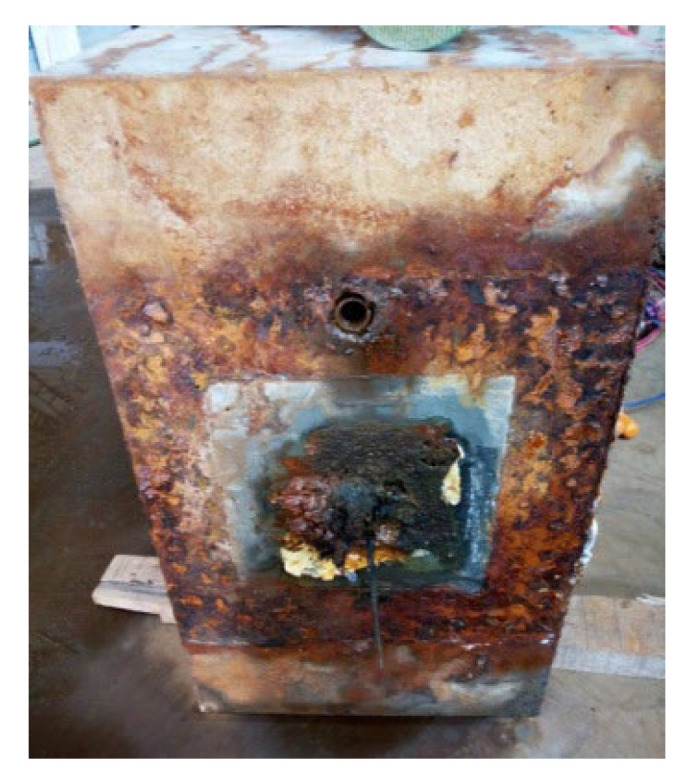
Corrosion fracture at end anchorage part of steel strands in test members.

After grouting and maintaining for 28 d, the anchorage zone at the ends of S1–S5 was locally corroded, and the corrosion mode was indoor electrochemical rapid corrosion. In this experiment, a local corrosion tank with a length of 20 cm was designed and fixed in the end area of concrete specimens using structural adhesive, and the tank was filled with 5% NaCl solution and installed with stainless-steel plates. During corrosion, the anode wire of the constant DC power source was turned on and connected to the steel strand, and the cathode wire of the power source was connected to the stainless-steel plate placed in the corrosion tank. At the same time, a current loop could be formed by pouring NaCl solution into the tank. Under the action of current, the anodic steel strand was corroded, as shown in Figure 2. During the test, only the corrosion at the end of the steel strand was considered. To avoid the influence of corrosion on ordinary steel bars, anti-corrosion treatment was performed by smearing epoxy resin when binding the ordinary reinforcement cage. The whole test was carried out in an environment with a temperature of 20 °C and humidity of 65%. It took about 5 d for the corrosion of the member. Through inspection, all prestressed tendons experienced corrosion fracture at the end, which was consistent with the test design, as shown in Figure 3.

When specimen S3 was tensioned and anchored, a metal block was inserted between the anchor cup at one end and the specimen. After 48 h from completing the tensioning, the metal block was removed to achieve direct release tensioning.

### 2.4. Pull-Out Test Design of Steel Strands and the Test Devices

**Figure 4:**
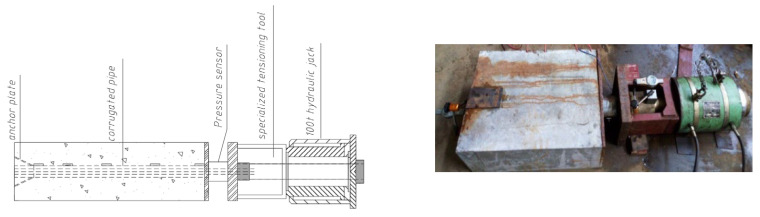
Pull-out test device.

To discuss the secondary anchorage test, the pull-out test was implemented on the uncorroded side after the corrosion fracture of the steel strands on one side of the member. For this purpose, connectors, pull rods, and anchorage devices were specially designed, as displayed in Figure 4. According to the rules of the concrete and steel strand grip test provided in the Testing Code of Concrete for Port and Waterway Engineering (JTS/T 236-2019) [27], the pull-out test device was self-designed. After completion, the test specimens were tensioned using a hydraulic jack with a range of 100 t, and the pull-out force was recorded with a pressure sensor.

The steel strands were pre-tensioned before the formal pull-out test to eliminate the nonelastic deformation between test devices, and the pre-tensioning force was set to 5 kN. After pre-tensioning, the readings of the anchorage dynamometer and dial indicator were reset at the loading end, and the formal loading procedure was started. The load was controlled as per the load staging: initially, a 5 kN load was applied at each stage and 15 kN was applied at each stage after reaching 15 kN. Meanwhile, various test phenomena such as the displacement at the loading end and drawing end of the members, the readings of the anchorage dynamometer and suspension-type strain gauge, crack development, and the test sound under various loads were recorded. The pull-out test would be stopped immediately under any of the following circumstances during the test process: (1) the steel strand was pulled out or experienced fracture failure; (2) the slip amount at the loading end exceeded 30 mm, the load could not be continuously increased or the increment was very small, and the bond–slip failed.

## 3. Results and Discussion

### 3.1. Failure Mode

**Figure 5:**
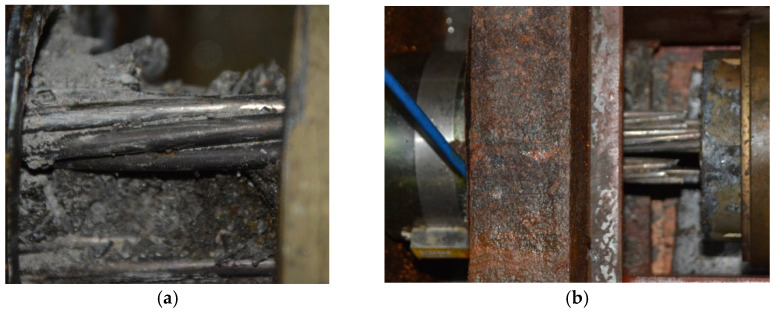
Failure modes of specimens: (**a**) bond–slip failure; (**b**) material tensile failure.

In this test, typical failure modes were observed in two categories: the first involved the steel strands, which were pulled out as a whole, resulting in bond–slip failure. However, it differed slightly from the individual strand slip process, in which three steel strands along with the concrete between them were entirely pulled out. The second category involved the fracture of steel strands resulting in material failure, as depicted in Figure 5. Specimens S1, S3, and S4 experienced bond slip failure, while specimens S5 to S7 exhibited material failure, as indicated in Table 4.

### 3.2. Crack Distribution

**Figure 6:**
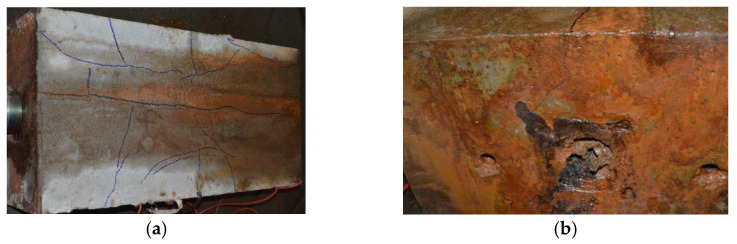
Crack diagram under ultimate state of type I specimens: (**a**) distribution of splitting cracksl (**b**) shape of the main splitting crack at the corrosion end.

Different test specimens exhibit varying crack distributions, which can be classified into three types based on the pattern of cracks. For the first type, splitting cracks occurred in specimens S1, S3, and S4. As the pull-out load continued to increase, a new crack along the direction of the steel strands started appearing at the tensioning end. When the load further increased to about 50% of the ultimate load, the initial tensile crack width started increasing from the pull-out end to the free end, and several secondary splitting cracks along the length direction appeared beside the splitting cracks; moreover, the free end started slipping. As the load increased near the ultimate load, the main and secondary splitting cracks both ran through the whole specimen, accompanied by a very loud splitting sound. Subsequently, the steel strands were pulled out when the load reached the ultimate value, as shown in Figure 6.

**Figure 7:**
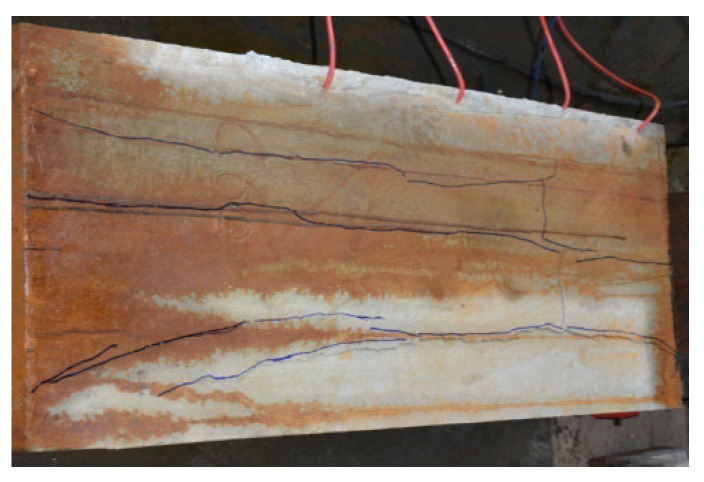
Crack diagram under ultimate state of type II specimens.

For the second type, longitudinal initial cracks appeared after the initial tensioning of S5 was completed. During the test, the width of the initial tensile cracks increased subtly with the increase in the pull-out load; moreover, the pull-out force reached about 60% of the ultimate pull-out force, several longitudinal cracks were added (but only one splitting crack appeared when the ultimate load was reached), and all cracks did not completely run through the whole specimen. As shown in Figure 7, the load reached the limiting value, and a loud sound was heard; moreover, the steel strands were ruptured, leading to material failure that was accompanied by the rotation of the jack, which might be associated with the twists that formed in the steel strands.

**Figure 8:**
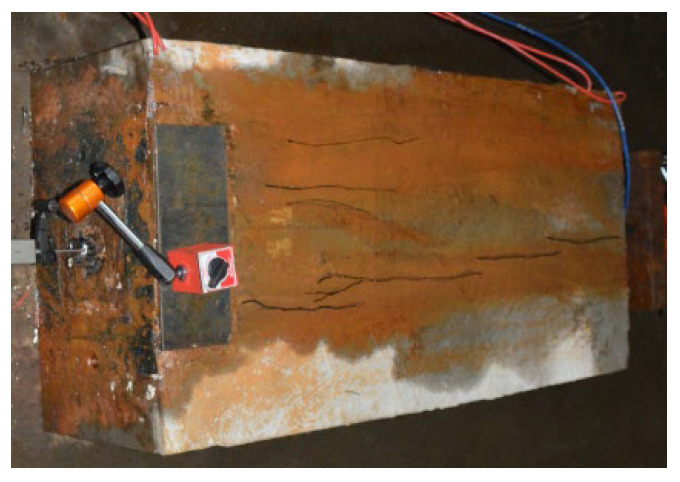
Crack diagram under ultimate state of type III specimens.

The third type of cracks occurred in S6 and S7. No secondary splitting cracks were found in such specimens during the whole test process. Compared with type II specimens, there were more longitudinal cracks; however, the length was smaller. A loud sound was heard when the load reached the limiting value: the steel strands were ruptured, and material failure occurred, as shown in Figure 8.

### 3.3. Load Bond–Slip Curves

**Figure 9:**
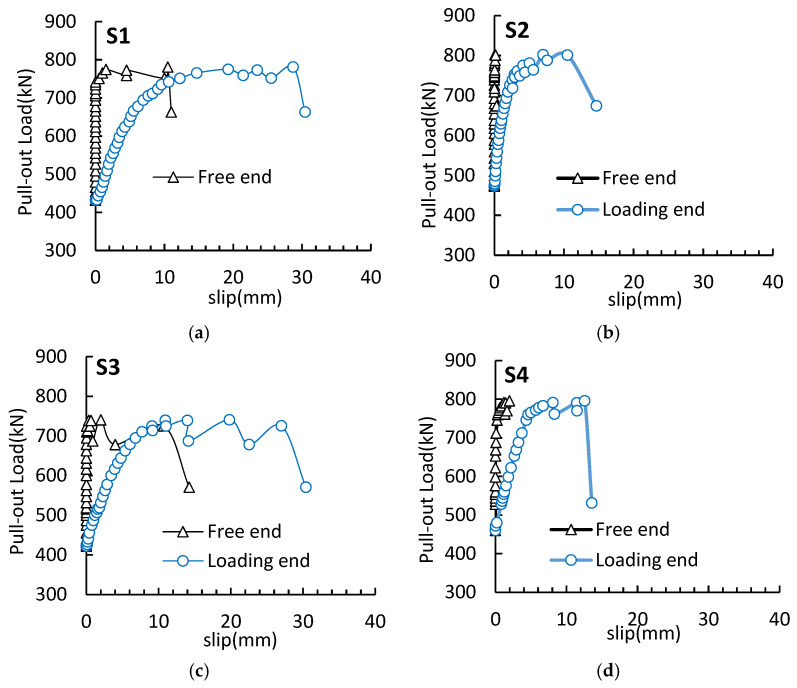

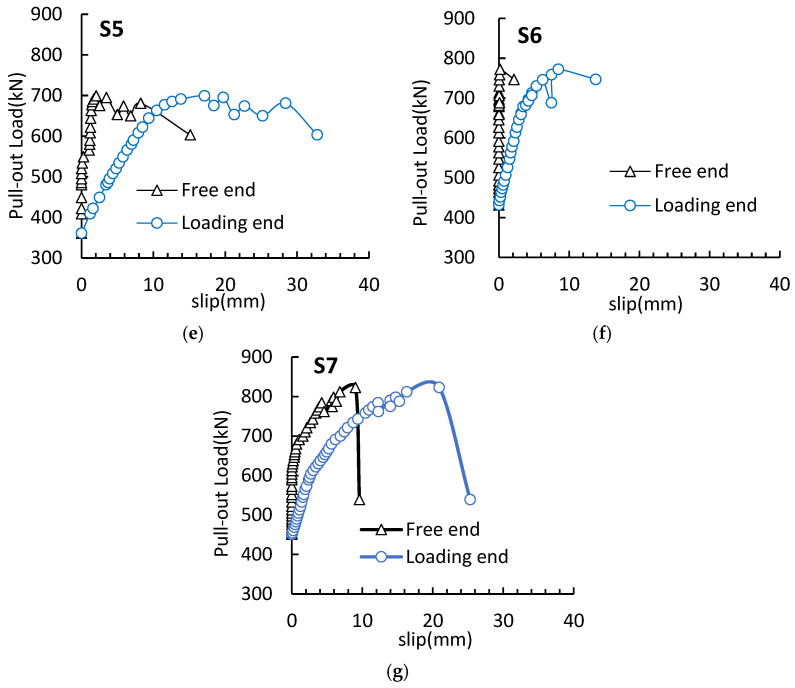
Load bond–slip curves at the loading end: (**a**) S1; (**b**) S2; (**c**) S3; (**d**) S4; (**e**) S5; (**f**) S6; (**g**) S7.

The load bond–slip curves between the loading end and the free end of specimens S1–S7 under the pull-out force are exhibited in Figure 9. For the same specimen, the loading end slipped earlier than the free end, and the growth rate and limiting value of the slip amount were both greater than those at the free end. This was because, during the stress transfer of the pull-out force inside the specimen, stress loss would occur, and the pull-out force borne at the free end was much smaller than that at the pull-out end.

It could be known from the load bond–slip curves on the loading side that the secondary anchorage failure process of the steel strands could be divided into three stages. The first was the linear stage: when the tensile force was less than about 30% of the ultimate pull-out load, the slip amount at the loading end was basically linear with the pull-out load, and the sliding friction between steel strands and concrete played a major role; the second was the yield stage: when the pull-out load continued to increase to about 90% of the ultimate pull-out load, the slip growth of the pull-out end was obviously accelerated, and the radial component of the mechanical bite force between the steel strands and the grouting body continued to increase with the increase in the slip amount. Meanwhile, the bonding force between the steel strand and the concrete was gradually destroyed, the free end began slipping, and the stress of the steel strands gradually increased, entering the yield stage; the third stage was the failure stage: the pull-out force increased slightly, and the slip value increased rapidly. The steel strands were in the post-yield stage, the pull-out force suddenly decreased and then increased again, and the pull-out force fluctuated in a certain range until the steel strands were finally broken. Relative to the type II specimens in which steel strands were broken, for the type I specimens experiencing the pull-out failure of steel strands, the ultimate slip value at the pull-out end exceeded 30 mm upon failure, and the specimens failed to provide enough secondary anchorage strength.

### 3.4. Influencing Factors

**Figure 10:**
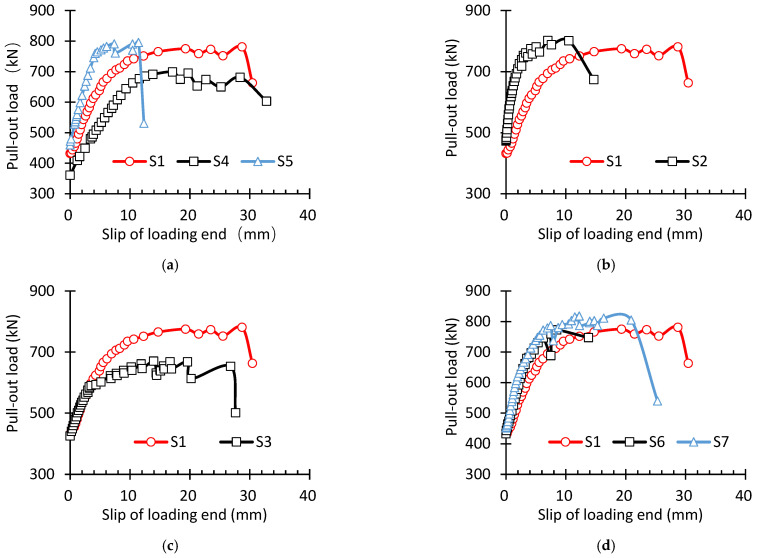
Comparison of load bond–slip curves under different test conditions: (**a**) influence of concrete strength on pull-out performance; (**b**) different specimen lengths; (**c**) different release-tensioning speeds and modes; (**d**) different stirrup configurations.

In order to study the influence of different parameters on the bond anchorage performance of specimens, Figure 10 shows the load slip curves at the loading end under the influence of different specimen lengths, prestress release methods, concrete strength, and stirrup diameters. Figure 10a shows the pull-out test results of specimens with different concrete strengths. Comparing the data in Figure 10 and Table 4, the ultimate pull-out force of specimen S1 was 747 kN, which was 10.3% lower than that of specimen S4 and 5.8% higher than that of S5. Under the ultimate load, the relative displacement between the pull-out end and the free end was 16.1 mm for S1 and 16.5 mm for S4, which was 2.5% higher than that for S1. The relative displacement for S5 was 10.5 mm, which was 34.8% less than that for S1. It could be seen that concrete strength had a significant influence on the pull-out performance of the specimens. The higher the concrete strength, the greater the ultimate pull-out force. Moreover, compared with the slippage and pull-out as a whole, the slip value at the loading end upon the tensile failure of the steel strands was significantly reduced, and the specimen stiffness was strengthened by high concrete tensile strength. This was because, the higher the concrete strength, the more obvious the radial squeezing effect on steel strands, increasing the circumferential tensile stress and delaying the appearance of both microcracks and splitting cracks in the specimen. Therefore, both the bond strength and stiffness of specimens significantly increased with the increase in concrete strength, and S1 and S4 developed from the first type of failure mode into the second type and experienced sufficient secondary anchorage.

Figure 10b shows the pull-out test results of specimens with different lengths. Compared with specimen S1, the length of S2 only increased by 250 mm. Comparing the data in Figure 10b and Table 4, the ultimate pull-out load of S2 increased by 5.8% compared with that of S1. The maximum slip amount of S2 was only 48.1% of that of S1. This is because, when the embedded length of the specimen was greater than the secondary anchorage length, the effective bond length could be increased by increasing the specimen length, the actual bond strength was smaller than the ultimate bond strength, and the overlapping between the length influenced by the pull-out force and the transfer length of residual prestress at the corrosion end was delayed, the development of splitting cracks was also delayed, and, thus, specimen stiffness grew evidently. The ultimate bond force provided by the specimen was directly proportional to the embedded length of steel strands. Hence, when the embedded length of the steel strands was greater than the secondary anchorage length, the failure mode in the pull-out test was transformed from the first type—slippage failure as a whole—into the second type—material failure induced by the rupture of steel strands.

Figure 10c shows the test results of different release-tensioning methods. Both S1 and S3 experienced pull-out slippage failure as a whole. Compared with S1, the ultimate pull-out load of S3 was reduced by 10.3%, and both specimens were subjected to the slippage failure of steel strands as a whole. In the initial loading stage, almost no differences were found between S1 and S3. As the load increased, the stiffness of S3 gradually declined because the specimen needed a greater secondary transfer length due to sudden release tensioning, which led to a reduction in the effective bond length. Under the pull-out force, the pull-out bond length of the specimen overlapped with the secondary transfer length earlier; moreover, the concrete splitting cracks also developed earlier, and both the structural stiffness and ultimate bond strength decreased.

Figure 10d shows the pull-out test results of specimens with different stirrup configurations. As more stirrups were configured, both S6 and S7 experienced the tensile failure of steel strands, indicating that the increase in the stirrup ratio facilitated sufficient secondary anchorage. Comparing the data in Figure 10d and Table 4 shows that, compared with S1, the ultimate pull-out force of S6 increased by 3.3% while that of S7 grew by 9.5%. The pull-out performance of the specimens was significantly affected by concrete strength: the higher the concrete strength, the greater the ultimate pull-out force. In comparison with S1, S7 possessed a longer platform after the yielding of steel strands. This was because, with the increase in the diameter of the stirrups, the horizontal restraining effect provided by the stirrups was enhanced, which could effectively restrain and delay the development of splitting cracks, prevent the splitting failure of specimens, increase the structural bond strength, and enhance structural ductility. Comparing the load–slip curves of S1, S6, and S7, however, the difference among the three specimens in the slope of the load–slip curves was not evident in the initial loading stage, and the structural stiffness could not be significantly increased by increasing the stirrups. This was because the stirrup stress was relatively small in this case; moreover, the average stress of concrete within the wrapping scope of stirrups was small, and the horizontal restraining effect of stirrups was also weak, thus exerting a limited effect when increasing the structural initial stiffness.

## 4. Numerical Simulation Analysis

### 4.1. Modeling

To further explore the secondary anchorage performance of residual prestress after the end anchorage failure of the post-tensioned, prestressed members, the finite element calculation model of damaged concrete specimens was established using Abaqus (version of Abaqus CAE2016). The structure was established by using a discrete model, and the reduced integral hexahedral element (C3D8R) was used to simulate concrete. The size of the concrete unit is 20 mm × 18.75 mm × 20 mm.

**Figure 11:**
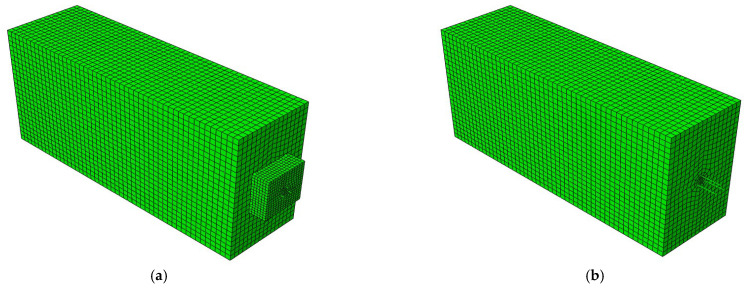
Overall structural model: (**a**) step 4 model; (**b**) step 6 model.

In the modeling process, a constitutive concrete damage plasticity (CDP) model was adopted. Regarding the model parameter of the CDP model, the measured value of the cubic compressive strength of the concrete was used. The tensile strength, elastic modulus, and other parameters of concrete were calculated according to specification GB50010-2020 [16]. The values of other CDP model parameters are shown in Table 5. Additionally, both ordinary steel bars and prestressed steel bars were simulated using truss elements (T3D2), and a constitutive relation model of double-broken-line, equal-strength hardening was selected [28]. A total of 28,522 nodes and 25,723 elements were set in the model, and the overall structural model is displayed in Figure 11.

To consider the bond slip between the steel strand and concrete, two different nodes are set at the same positions as the steel strand and concrete, which are connected using nonlinear spring elements (SPRING2), and the bond–slip constitutive model in [29] is transformed into a spring constitutive model. In addition, the anchor pad is connected using the tie-in interaction module, and the connection between ordinary steel bars and concrete is embedded. To be consistent with the constraints in the actual working conditions, three boundary conditions, DX, DY, and DZ, are added to the constrained concrete element in the actual working conditions. By activating and passivating the boundary conditions, the constraint changes in the prestressed tension stage, corrosion stage, and drawing stage are simulated.

The external loads that need to be applied during the simulation process mainly include prestress and tensile force. The prestressing force is applied using the cooling method, while the pull-out load is applied to steel strand nodes through the direct loading method. The external loads that need to be applied during the simulation process mainly include prestress and tensile force. The prestressing force is applied using the cooling method, while the pull-out load is applied to the steel strand nodes through the direct loading method.

The static and general analysis steps were adopted as follows: In step 1, all structural elements were inactivated. In step 2, the concrete elements, anchor plates, steel strands, reinforcement cages, and boundary conditions beyond the grouting part were activated. In step 3, prestress was applied through the cooling method. In step 4, grouting elements were activated. In step 5, the steel strand elements and anchor plates at the fracture part were inactivated. In step 6, the anchor blocks on the pull-out load side were inactivated, and the pull-out load was applied to the steel strand nodes.

### 4.2. Verification of Calculation Results

Because static and general analysis steps were adopted, only S1–S2 and S3–S7 were subjected to the finite element method. The calculated and measured values of the ultimate pull-out load for the specimens are listed in Table 6. It could be seen that the relative error between the test value and the numerically calculated value of the ultimate pull-out load of each specimen was relatively small, with the maximum value only being 5.0%, and all calculated values relatively coincided with the test values.

**Figure 12:**
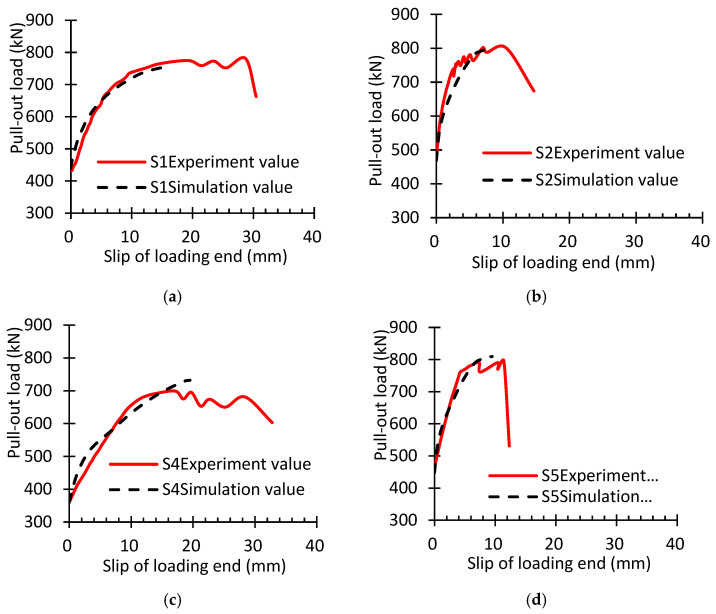

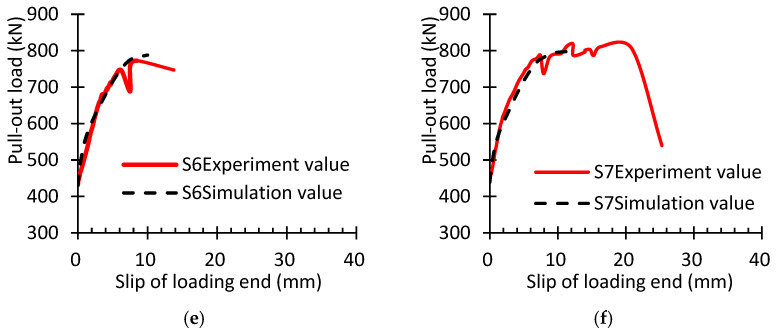
Comparison between test results and calculation results of load bond–slip curves: (**a**) S1; (**b**) S2; (**c**) S4; (**d**) S5; (**e**) S6; (**f**) S7.

See Figure 12 for the test value and numerically calculated value of the loading–end slip value of different specimens under different pull-out loads. It could be observed that the test value of the loading–end slip amount under different loads was relatively consistent with the calculated value, further verifying the effectiveness of the calculation model. Figure 12b needs to be adjusted to the following figure.

**Figure 13:**
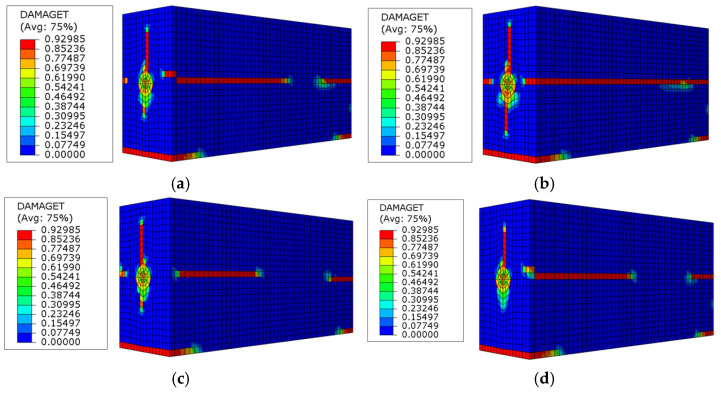

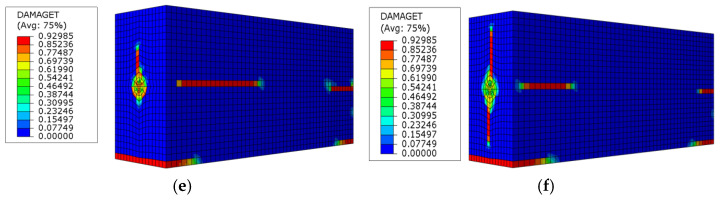
Comparison of tensioning-induced concrete damage and cracking of specimens under the pull-out load of F = 730 kN: (**a**) S1; (**b**) S2; (**c**) S4; (**d**) S5; (**e**) S6; (**f**) S7.

In order to compare the damage status of concrete under the same level of the pull-out load and the secondary anchorage status of residual prestress in the specimens, the influence of the embedded length of steel strands, concrete strength, and stirrup configuration on the secondary anchorage of residual prestress was analyzed theoretically. Referring to the ultimate bearing capacity of each specimen, the pull-out load was 730 kN, and the concrete damage and cracking induced by tensioning and the stress state of concrete in each specimen are shown in Figure 13. It can be seen from Figure 13 that, when the stress of the steel strands reached 1730 MPa, splitting cracks appeared in all specimens, and one to four secondary splitting cracks were produced. Therein, the main splitting cracks already ran through the whole S4 specimen. The increasing embedded length of the steel strands could postpone the generation of the main splitting cracks; however, the effect was not evident in the free-end secondary splitting cracks. Increasing the concrete strength and the stirrup ratio could effectively constrain the generation of splitting cracks and enhance the splitting stress and ultimate bond strength of specimens, which coincided with the test results and further verified the effectiveness of the calculation model.

## 5. Conclusions

(1) After the end anchorage failure of post-tensioned, prestressed specimens, the secondary anchorage bond performance of residual prestress is affected by factors such as the embedded length of steel strands, release-tensioning speed, concrete strength, and stirrup ratio. The increase in the embedded length of steel strands and the strength grade of concrete is beneficial for improving the bonding performance of the specimens;
(2) The pull-out process of prestressed specimens can be divided into three stages: the linear stage, yield stage, and failure stage. After the maximum pull-out force is reached, the specimen experiences bond failure or fracture failure in the prestressed steel strands;
(3) In this study, the construction condition considered is the corrosion fracture of end steel strands as a whole, which can be accompanied by corrosion damage along the full length of steel strands; however, this factor is not taken into account;
(4) The specimen sizes are evidently smaller than those in actual engineering structures, and the number of specimens is small. In addition, the secondary anchorage bond performance of residual stress after the end corrosion fracture of steel strands in practical engineering remains to be further investigated.

## Figures and Tables

**Table 1:** Parameters of specimens S1–S7.

Specimen No.	Sectional Dimension (mm)	Length (mm)	Concrete Grade	Stirrup Diameter (mm)	Fracture Mode
S1	300 × 500	1000	C50	Φ8	Corrosion-induced fracture
S2	300 × 500	1250	C50	Φ8	Corrosion-induced fracture
S3	300 × 500	1000	C50	Φ8	Direct release-tensioning
S4	300 × 500	1000	C40	Φ8	Corrosion-induced fracture
S5	300 × 500	1000	C60	Φ8	Corrosion-induced fracture
S6	300 × 500	1000	C50	Φ10	Corrosion-induced fracture
S7	300 × 500	1000	C50	Φ12	Corrosion-induced fracture

**Table 2:** Material properties of steel strands and ordinary steel bars.

Diameter (mm)	Yield Strength (MPa)	Ultimate Strength (MPa)	Elastic Modulus (GPa)
15.2	1810	1915	195
12	476	612	200
8	263	366	210
10	285	357	210

**Table 3:** Effective prestress in each test stage.

Specimen No.	S1	S2	S3	S4	S5	S6	S7
Tensioning prestress (kN)	636.12	636.12	636.12	636.12	636.12	636.12	636.12
Effective prestress before the corrosion test (kN)	497.2	511.3	499.5	449.9	510.5	501.1	505.2
Effective prestress after the anchorage failure (kN)	497.2	511.3	426.3	449.9	510.5	501.1	505.2

**Table 4:** Ultimate bearing capacity in pull-out tests of specimens S1–S7.

Specimen No.	Failure Mode	Initial Tensile Force of Steel Strands (kN)	Ultimate Tensile Force of Steel Strands (KN)	Pull-Out Force (kN)	Ultimate Slippage at Pull-Out End (mm)	Ultimate Slippage at Tensioning End (mm)	Crack Type
S1	Pull-out as a whole	432	765	315	14.5	30.6	Type I
S2	Rupture	456	802	345	0.3	10.5	Type II
S3	Pull-out as a whole	425	670	245	15.1	32.5	Type I
S4	Pull-out as a whole	361	695	335	15.2	31.7	Type I
S5	Rupture	460	793	333	2.0	12.5	Type II
S6	Rupture	451	772	321	2.2	13.8	Type III
S7	Rupture	431	818	387	9.1	20.9	Type III

**Table 5:** CDP model parameters.

Poisson’s Ratio	Expansion Angle	Eccentricity	Parameters Affecting the Yield Morphology of Concrete	Ratio of Ultimate Strength under Biaxial and Uniaxial Compression	Viscosity Coefficient
0.2	30	0.1	0.667	1.16	0.005

**Table 6:** Comparison between test values and calculated values of ultimate bearing capacity in the pull-out test.

Ultimate Load (kN)	Specimen No.					
	S1	S2	S4	S5	S6	S7
Test value	771	802	695	793	772	818
Calculated value	753	791	730	809	783	797
Relative error (%)	2.3	1.4	5.0	2.1	1.4	2.5

## Data Availability

Data are contained within the article.
